# Substandard and falsified medical products: bibliometric analysis and mapping of scientific research

**DOI:** 10.1186/s12992-021-00766-5

**Published:** 2021-09-23

**Authors:** Waleed M. Sweileh

**Affiliations:** Department of Physiology, Pharmacology/Toxicology, College of Medicine and Health Sciences, An-Najah National University, Nablus, Palestine

**Keywords:** Counterfeit drugs, Substandard medications, Falsified drugs, Research activity, Bibliometric analysis

## Abstract

**Objective:**

Substandard and falsified (SF) medical products are a global public health threat. The presence and spread of SF drugs negatively affect (1) patients’ safety and health outcomes, (2) national economy, (3) public trust in the healthcare system, and (4) the international fight against serious health challenges such as malaria and antimicrobial resistance. The objective of the current study was to investigate and provide a snapshot analysis of the evolution and developmental patterns of global research publications on SF products.

**Methods:**

A bibliometric approach was adopted using terms such as fake, falsified, counterfeit, substandard, and others. No language restriction was made. The study period was from 1900 up to 2020. The search strategy was validated and implemented using Scopus database.

**Results:**

The search strategy retrieved 978 documents authored by 2861 researchers from 100 different countries and published in 421 different journals. The retrieved documents received 11,237 citations (11.5 citations per document) with an H-index of 53. The 978 documents retrieved from Scopus were published from 1961 to 2020, giving an average of 16.6 publications per year. The present study indicated that research on SF medical products: (a) has experienced a steep growth from 2001 to 2012 followed by a steady-state growth; (b) was disseminated in a wide range of journals, mainly in the fields of the pharmaceutical industry, analytical chemistry, public health, infectious diseases, and internal medicine; (c) was published by scholars with diverse and distant geographical backgrounds; (d) was mainly produced in the United States, United Kingdom, and Germany; (d) has fragmented research networks and a limited number of researchers per network; (e) has limited cross-country collaboration except for that between the US and the UK in one hand and countries in the Mekong region in the other hand; (f) emphasized on medications related to malaria and sexual stimulants; and (g) received relatively inadequate funding.

**Conclusions:**

Research on SF medical products is important and should remain a priority to ensure good quality of medications. Research activity in the field needs to be encouraged in world regions such as Africa and the Middle East where drug regulations are unsatisfactory and cross-border trade of illegal medications is common.

**Supplementary Information:**

The online version contains supplementary material available at 10.1186/s12992-021-00766-5.

## Background

Falsified medicines, also known as counterfeit or fake medicines, are medical products that are deliberately and fraudulently mislabeled with respect to identity, composition, or source [1]. Substandard medicines (also called “out of specification”) are legal pharmaceutical products that fail to meet either their quality standards or specifications or both [1]. The global health challenge of ‘counterfeit’ medicines was first addressed in 1985, at the *Conference of Experts on the Rationale use of Drugs* in Nairobi. Subsequently, the *World Health Assembly Resolution* (WHA41.16; 1988) requested the initiation of programs for the prevention and detection of “falsely labelled, counterfeited or substandard pharmaceutical preparations”. In 1992, the *World Health Organization* (WHO) held a special conference on counterfeit drugs followed by the release of the “WHO Guidelines for the Development of Measures to Combat Counterfeit Drugs” [2]. Under the auspices of the WHO, the International Medical Products Anti-Counterfeiting Taskforce (IMPACT) was launched in 2006 to establish coordinated networks to fight counterfeit medicines [3].

On 29 May 2017 at the Seventieth World Health Assembly, a decision was agreed to adopt the “Substandard and Falsified (SF) medical products” term and to drop the term counterfeit drugs [4]. Substandard and falsified medical products include branded and generic pharmaceuticals, correct or wrong or no active ingredients, incorrect quantities of active ingredients, or fake packaging [1]. The term counterfeit is still used within the intellectual property legislation about consumer goods where counterfeit products such as clothes or shoes tend to have fake company logos resulting in patent or trademark infringement [5].

The presence of SF medical products is a growing wicked problem and a crime against humanity. The United Nations Sustainable Development Goals for 2030 emphasizes the “safe, effective, quality and affordable essential medicines” acknowledging the serious impact of fake medicines on global health [6]. The difficulty in controlling the market of falsified medicines can be attributed to the complex distribution network of medicines [7] as well as the profitable business of the falsified medicines [8]. The SF medical products are mostly found in countries where access to good quality medicines is limited or implementation of good pharmaceutical manufacturing practices is poor or absent [9]. A systematic review of prevalence studies carried out in 25 Low- and Middle-income countries found that the median prevalence of counterfeit medicines was 28.5 % (11–48.0 %) [10]. However, the globalization of the pharmaceutical market and the rise of internet use and online sale of various types of medical products led to the spread of SF medical products in developed countries [11]. The SF medical products negatively affect global health and strengthen international criminal networks [12]. The SF medical products included all types of medicines ranging from lifestyle products to life-saving ones. However, antibiotics and antimalarials were the most commonly reported ones.

Research activity on SF medical products is important nationally and internationally to help in the fight against SF medical products. Research in this field could help in developing governmental policies for screening and detection of reported SF products resulting in the future decline of incidents about SF products. The total number of counterfeit incidents concerning pharmaceuticals worldwide from 2002 to 2019 increased from 196 incidents in 2002 to more than 5000 incidents in 2019 [13]. This number would have been much higher in the absence of adequate regulations and policies Research on SF products is very important in identifying the stage at which the SF products were falsified. This will help governments to establish policies about anti-falsifying strategies targeting different levels of the pharmaceutical supply chain [14].

Research in this field helps in increasing awareness of healthcare professionals and health policymakers about the presence of such a wicked problem. Second, research activity in this field enhances the momentum of research directed to the development of quick and reliable methods for the detection of SF medical products. Third, research activity in this field is in agreement with the WHO recommendation for the importance of availability and access to high-quality medicines as a basic human right. Fourth, research activity in this field helps Interpol and other agencies in fighting pharmaceutical crime and trafficking of SF medical products. Fifth, research in this field helps in developing national regulations about monitoring pharmaceutical manufacturing and marketing pharmaceuticals in any country. Given all this, it is important to assess the global research activity on SF medical products to analyze patterns and hotspots in the field and to add new information to the field.

The seriousness and global dimension of the SF medical products necessitate the collection of literature on the topic and analyzing it quantitatively. Such analysis is highly facilitated by the presence of large academic databases such as Scopus or Web of Science. The use and application of statistical methods on scientific research publications retrieved from academic databases to measure research activity of countries, authors, institutions, journals, and to assess the growth pattern of publications over time is termed bibliometrics [15]. Bibliometric analyses are carried out for several reasons such as to (1) uncover the emerging trends of publications in a certain field, (2) find the extent of research collaboration, (3) explore the intellectual structure of a specific scientific field, (4) identify knowledge gaps, and (5) find novel ideas for future investigations. Results of bibliometric analysis usually provide evidence for policymakers to optimize research activity. Based on the author’s best knowledge, no bibliometric analysis of literature on SF medical products has been conducted. Therefore, the current study aimed to characterize the current landscape of literature on SF medical products published in peer-reviewed journals. The current study is not a systematic or narrative or a scoping review. There is a difference between bibliometric analysis and other types of reviews. For example, a scoping review is a type of knowledge synthesis in which the main focus of the analysis of the relevant literature is on the study design to synthesize evidence for future research designs [16]. A systematic review is a type of evidence synthesis that uses repeatable analytical methods to collect secondary data and analyze it [17]. Narrative reviews have broader research questions than systematic reviews. In systematic reviews, the selection criteria and processes are usually published whereas in narrative reviews the selection criteria and processes are not known to the reader [18].

## Methods

The current study is a bibliometric descriptive analysis of documents published on SF products.

### Database

In the present study, Scopus database was used to retrieve relevant documents on SF medical products because of the advantages it has over other scientific databases. Scopus is not 100 % ideal but it is 100 % inclusive of PubMed, has more than 23 thousand indexed journals in all scientific fields, has more documents than Web of Science, and has all functions needed for bibliometric analysis.

### Search strategy

The search strategy was developed to retrieve relevant documents published from 1900 to December 31, 2020. Several systematic reviews on the topic were examined to find all relevant terms [19, 20]. The terms used in the search strategy are shown in Appendix 1. Examples of the terms and phrases used included fake or substandard or falsified or *counterfeit or falsification or counterfeiting or “low quality” or “poor quality” in combination with one of the following terms in the title: medication or drug or medicine or pharmaceutical or antimicrobial or antimalarial or antibiotic or anti-infective or anticancer or “medicinal” or pharma*. The search strategy also included certain expressions such as “pharma* fraud” or “falsified medical product*” or “adulterated medicines” or “drug fraud” or “medication fraud” or “spurious medicine*”.

### Inclusion and Exclusion criteria

The search strategy focused on documents published in peer-reviewed scientific journals. Therefore, books and book chapters were excluded. Books were excluded because the content of most published books is a repetition or summary of published research articles. No language retraction was used in the search strategy. However, documents published in 2021 were not included because the year is not over yet at the time of analysis. In the search strategy, the quotation marks were used to retrieve the exact phrase while the asterisk was used as a wildcard. Appendix 2 shows the complete steps followed in the search strategy and the number of documents retrieved in each step.

### Validation

The search strategy was validated using two approaches. In the first approach, the top 100 cited documents were reviewed by checking the titles and abstracts and consulting two independent volunteers in case of doubt. This approach was carried out to fine-tune the search strategy and to eliminate terms that could retrieve false-positive results. The final search strategy produced no false-positive results confirming the validity of the search strategy. The second approach was to investigate two of the most active authors as obtained by the search strategy. In the present study, Newton, P.N. and Fernandez, F.M. were among the top active authors with 27 and 21 publications respectively. To confirm that the research strategy was comprehensive and retrieved all possible data, the research activity of the two researchers was investigated and counted using author search methodology in Scopus. The result of this approach showed that the two researchers have similar research output to that produced by the search strategy indicative of the high validity of the search strategy.

### Bibliometric analysis of core countries (top 10 active countries)

One of the bibliometric indicators presented in the current study was core (top ten active) countries and core authors involved in publishing documents on SF products. For each article, Scopus provides the list of all authors with his/her country affiliation. The analysis of core countries and core authors involved the same methodology applied by Scopus. For example, an article with three authors affiliated with three different countries was counted three times, once for each country. That is why there is an overlap in the numbers presented in the core countries. The same applies to authors with two different country affiliations. For example, an article with one author having an affiliation in the US and Thailand was counted twice; once for the US and once for Thailand. For core authors, the same approach was used. For example, an article authored by two researchers (X and Y) was counted twice, once for researcher X and once for researcher Y. Therefore, two researchers in the core list might have many common papers. This approach was used in all bibliometric studies regardless of the database used.

### Data export and management

The search strategy was implemented in the advanced search function in Scopus. The retrieved documents were analyzed using the “analyze” function. The results abstained were exported as csv Microsoft files. The results exported included annual growth, names of journals, names of authors, names of countries, and names of institutions. This information was presented in the result section as a linear graph of the annual growth of publication and as a table of core (top ten active) journals, authors, countries, and institutions.

The retrieved data was also exported to the online visualization program, VOSviewer for further analysis and visualization [21]. The map created included author keyword co-occurrence analysis to show major research topics found in the retrieved documents. Mapping also included cross-country collaboration and co-authorship analysis. In all visualization maps, the node size is proportional to the frequency and color of the node is a function of relatedness and the distance between nodes is a function of the strength of relatedness.

## Results

### Descriptive characteristics of the retrieved data

The search strategy retrieved 978 documents. The most common type of retrieved documents was research articles (n = 542, 55.4 %), followed by review articles (n = 139, 14.2 %), notes (n = 102, 10.4 %), short surveys (n = 77, 7.9 %), editorials (n = 50, 5.1 %), letters (n = 46, 4.7 %), and conference papers (n = 22, 2.2 %). There were 105 (10.7 %) documents with bilingual titles (German/English) and 28 (2.9 %) documents with French/English bilingual titles. Of all the retrieved documents, 257 (26.3 %) were published in open access sources while the remaining documents were published in non-open access (subscription) sources. In total, 2861 authors (mean = 2.9 authors per document) participated in publishing the retrieved documents. There were 451 (46.1 %) single-authored documents, 141 (14.4 %) two-authored documents, 93 (9.5 %) three-authored documents, 81 (8.3 %) four-authored documents, and the remaining 99 (10.1 %) were multiple-authored (≥ 5 authors per document) documents. The retrieved documents received 11,237 citations (11.5 citations per document) with an H-index of 53.

### Evolution and growth trajectory of publications and citations

**Fig. 1:**
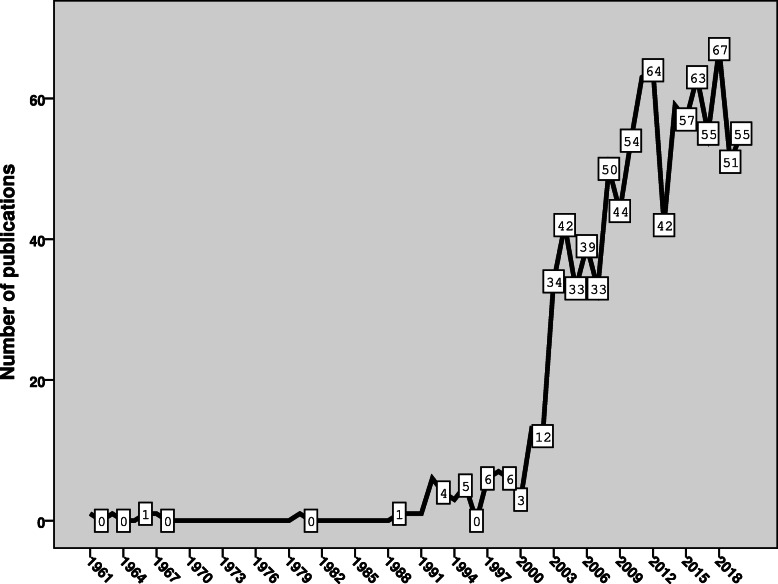
Annual growth of publications on SF medical products

The 978 documents retrieved from Scopus were published from 1961 to 2020, giving an average of 16.6 publications per year. The growth trajectory of publications has four distinct phases (Fig. 1): the first phase (1961–1991) in which the growth of publications was close to zero; the second phase (1992–2000) in which the growth showed the emergence of the field; the third phase (2001–2012) in which the growth of publications was characterized by a steep upward increase; and the final phase (2013–2020) in which the growth of publications showed a fluctuating steady state.

### Core Journals

The retrieved documents were published in 421 different journal names. Table 1 presents the top ten active journals (core list) in the field. The *Pharmazeutische Industrie* journal stands out as the main publication venue in the field, accumulating around 4.2 % (n = 41) of the publications in the dataset. However, documents published in the *Journal of Pharmaceutical and Biomedical Analysis* received the highest number of citations per document (30 citations per document). Most of the journals in the core list were in the field of pharmacy. Four of the journals in the core list published articles in the non-English language. However, there were three non-pharmacy journals in the core list: *The Lancet*, *BMJ Clinical Research Ed*, and the *American Journal of Tropical Medicine and Hygiene*. Approximately 27 documents were within the field of social sciences, specifically in the field of criminology, human rights, law, and intellectual property [22–26].

**Table 1:** Core journals publishing documents on substandard and falsified medical products

Rank	Journal name	Frequency	% (n = 978)	Citations per document
**1**	*Pharmazeutische Industrie*	41	4.2	0.9
**2**	*Journal Of Pharmaceutical And Biomedical Analysis*	38	3.9	30
**3**	*Deutsche Apotheker Zeitung*	26	2.7	0.1
**4**	*Lancet*	25	2.6	15.1
**5**	*BMJ Clinical Research Ed*	22	2.2	7.5
**6**	*American Journal Of Tropical Medicine And Hygiene*	19	1.9	20.1
**7**	*Pharmaceutical Journal*	18	1.8	1.1
**8**	*Pharmaceutisch Weekblad*	16	1.6	0
**9**	*Drug Topics*	14	1.4	0.1
**10**	*Pharmazeutische Zeitung*	13	1.3	0.1

### Leading authors, countries, and institutions

The 978 documents retrieved from Scopus were published by a total of 2861 authors from 100 countries worldwide. Table 2 shows the researchers with the highest number of publications in the field. Newton, P.N. and Fernández, F.M. appear as the most productive researchers. Four authors in the list were based in the US, three based in Belgium, three based in Germany, two based in the UK, and one has double country affiliations, in specific, the UK and Thailand.

**Table 2:** Core authors publishing documents on substandard and falsified medical products

Rank	Author Name	Frequency	% (n = 978)	Country Affiliation
**1**	Newton, P.N.	27	2.8	UK
**2**	Fernandez, F.M.	21	2.1	USA
**3**	Green, M.D.	18	1.8	USA
**4**	Deconinck, E.	16	1.6	France
**5**	Bate, R.	12	1.2	USA
**6**	Holzgrabe, U.	11	1.1	Germany
**7**	Mackey, T.K.	10	1.0	USA
**8**	Kaur, H.	9	0.9	UK
**8**	White, N.J.	9	0.9	UK/Thailand
**10**	Courselle, P.	8	0.8	Belgium
**10**	Hubert, P.	8	0.8	Belgium
**10**	Kimura, K.	8	0.8	Japan
**10**	Sacré, P.Y.	8	0.8	Belgium

Countries leading research on SF medical products are presented in Table 3. The US (n = 213, 21.8 %) is the leader in this field, publishing approximately double the number of publications from the UK (n = 117, 12.0 %) and approximately three times the number of publications produced in China (n = 29, 3.0 %) and India (n = 39, 4.0 %) combined. The list of core countries did not include any country in the African region or the Eastern Mediterranean region, or Latin America, or Eastern Europe.

**Table 3:** Core countries publishing documents on substandard and falsified medical products

Rank	Author Name	Frequency (all documents) (%, M = 978)	Frequency (research articles only (%, N = 542)	Frequency (Review articles only) (%, N = 139)	TLS
**1**	United States	213 (21.8)	113 (20.8)	48 (34.5)	100
**2**	United Kingdom	117 (12.0)	62 (11.4)	26 (18.7)	99
**3**	Germany	101 (10.3)	49 (9.0)	19 (13.7)	9
**4**	Belgium	54 (5.5)	31 (5.7)	11 (7.9)	28
**5**	France	41 (4.2)	26 (4.8)	8 (5.8)	18
**6**	India	39 (4.0)	23 (4.2)	6 (4.3)	19
**7**	China	29 (3.0)	24 (4.4)	2 (1.4)	13
**8**	Switzerland	27 (2.8)	14 (2.6)	7 (5.0)	19
**9**	Netherlands	20 (2.0)	11 (2.0)	4 (2.9)	21
**10**	Australia	19 (1.9)	6 (1.1)	6 (4.3)	18
**10**	Italy	19 (1.9)	12 (2.2)	2 (1.4)	8

*TLS* Total link strength. It is a measure of international research collaboration. The higher the TLS value, the higher the extent of international research collaboration. Values of the TLS were calculated and obtained directly from the VOSviewer program

At the institutional level, the *University of Oxford* (n = 39, 4.0 %) was the most productive, followed by *Georgia Institute of Technology* (n = 20, 2.0 %), and the US CDC (n = 19, 1.9 %). Table 4 shows the top 10 active institutions. The list included four institutions in the US, two institutions in the UK, three institutions in Belgium, and one institution in Lao. Half of the institutions in the core list were non-academic institutions.

**Table 4:** Core institutions publishing documents on substandard and falsified medical products

Rank	Author Name	Frequency	% (n = 978)	Country Affiliation
**1**	*University of Oxford*	39	4.0	UK
**2**	*Georgia Institute of Technology*	20	2.0	USA
**4**	*Centers for Disease Control and Prevention*	19	1.9	USA
**5**	*Mahosot Hospital, Lao*	17	1.7	Lao
**6**	*London School of Hygiene & Tropical Medicine*	15	1.5	UK
**7**	*Universite de Liege*	13	1.3	Belgium
**7**	*Food and Drug Administration*	13	1.3	USA
**9**	*American Enterprise Institute*	11	1.1	USA
**9**	*Universiteit Gent*	11	1.1	Belgium
**9**	*Scientific Institute of Public Health, Brussels*	11	1.1	Belgium

### Cross-country collaboration

**Fig. 2:**
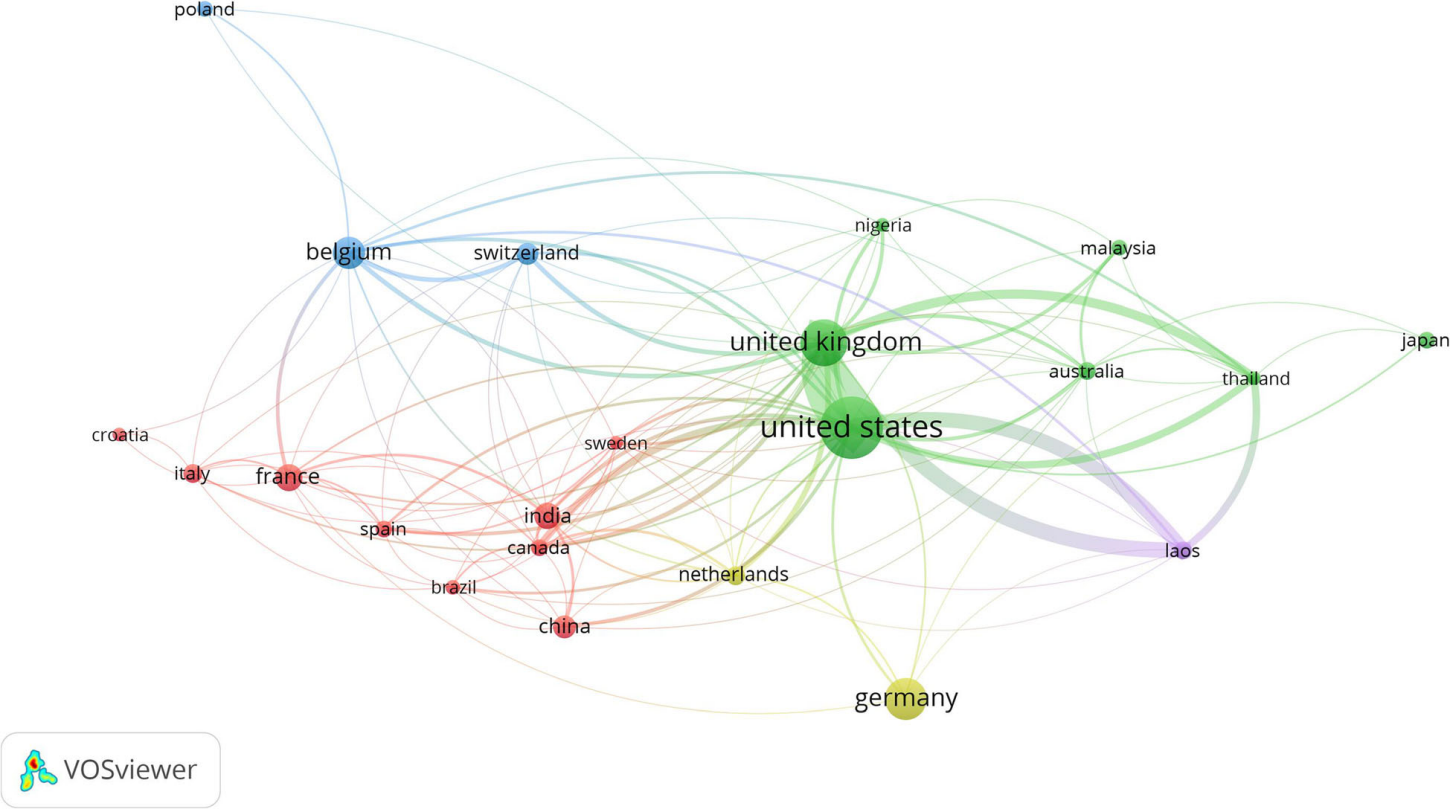
Network visualization map of international research collaboration between countries with a minimum contribution of 10 documents (n = 22). The thickness of the connecting lines represents the strength of collaboration

Cross-country network collaboration was presented in Fig. 2. The network included countries with a minimum contribution of 10 documents (n = 22). The map has four clusters representing four different cross-country network collaborations. The strongest cross-country collaboration, as measured by the thickness of the connecting line, was between the UK and USA, followed by that of UK-Lao, US-Lao, UK-Thailand, and US-Thailand. Other cross-country collaboration networks were relatively weak as measured by the thickness of the connecting lines. The US and the UK have research collaboration with low-income, non-English speaking, and geographically distant countries such as Lao, and Thailand. Both the US and the UK occupied the center of the map and had research collaboration with almost all countries on the map as shown by the connecting lines.

### Disciplines underlying the foundation of the field

**Fig. 3:**
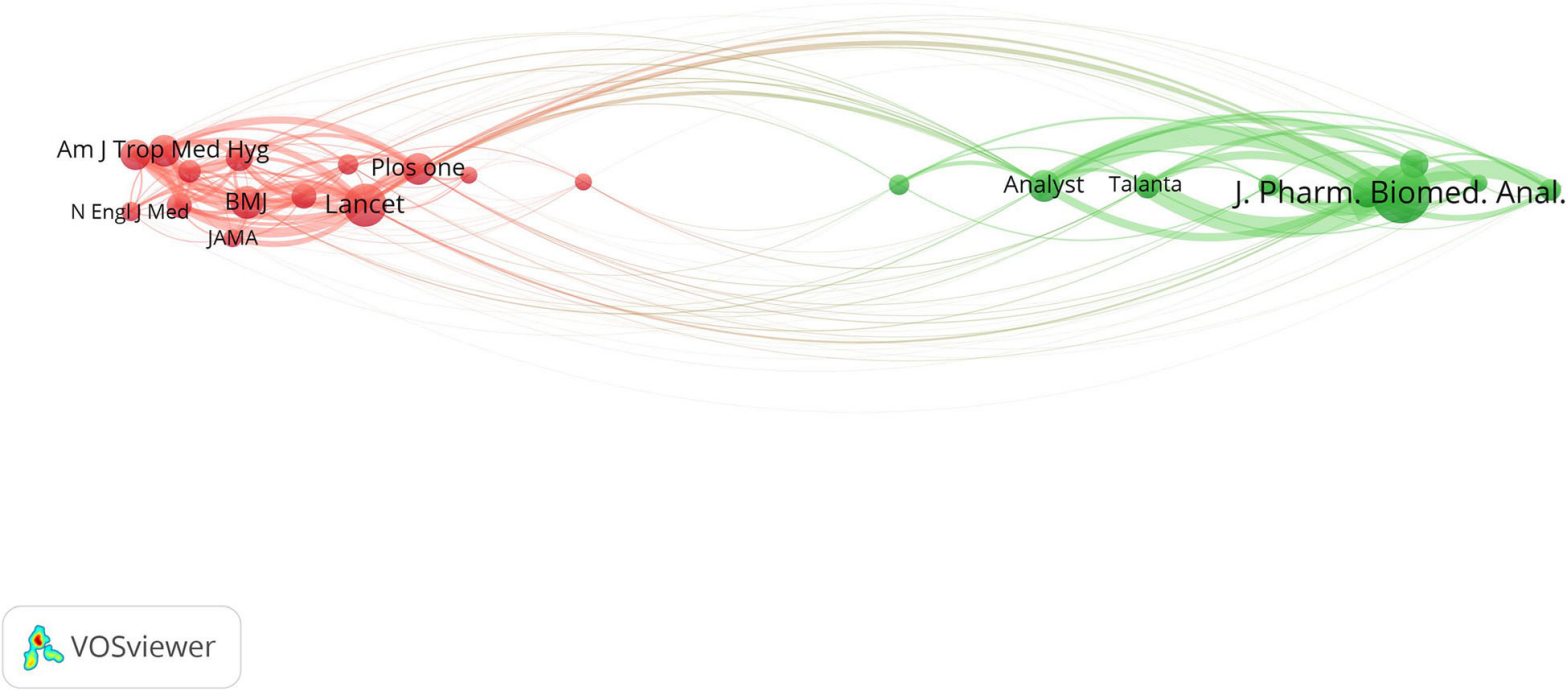
Co-citation analysis of journals with minimum citations of 50 (*n* = 23). Clusters represent the scientific disciplines underlying the emergence of the SF medical products scientific research

The journal co-citation analysis was performed. In this analysis, only journals with at least 50 citations were considered (*n* = 23). Figure 3 is a co-citation analysis of highly co-cited journals. Research on SF medical products mainly emerges from the convergence of research conducted in pharmaceutical analysis, public health, and infectious diseases. The red cluster integrates journals on medicine, public health, and infectious disease. The blue cluster represents the contribution of chemistry, chromatography, and pharmaceutical journals.

### Major topics addressed in the retrieved literature

**Fig. 4:**
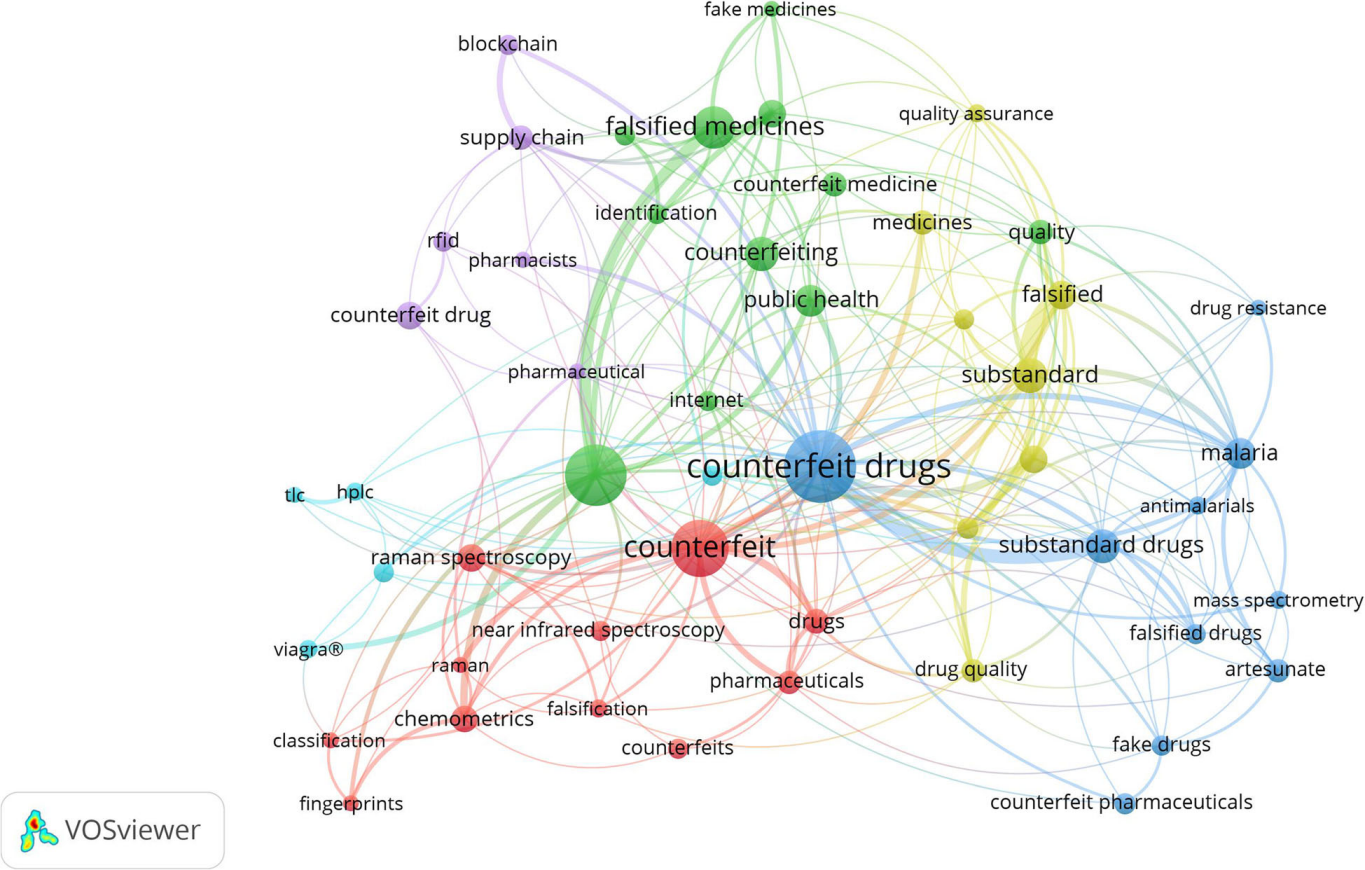
Network visualization map of co-occurrence analysis of author keywords with a minimum occurrence of five (n = 51). Different clusters represent major research topics encountered in the retrieved documents

Figure 4 shows the major topics of research on SF drugs during the past 60 years. The map is a co-occurrence analysis of author keywords. The map included author keywords with a minimum of five occurrences (n = 51). The size of the nodes indicates the frequency of occurrence of keywords while the thickness of connecting lines represents the co-occurrence strength between pairs of keywords. The most frequent keywords in the map including ones related to (a) malaria/antimalarial drugs (green cluster); (b) lifestyle medications such as the sexual stimulant; sildenafil (yellow cluster); (c) instrumental techniques used in the analysis of SF medical products (blue cluster); (d) health impacts of SF medical products and anti-counterfeiting technology (red cluster); and finally, (e) pharmacovigilance concerning SF medical products (purple cluster).

### Top 10 impactful documents

The top-cited documents reflect topics that are considered hotspots in the field and received the highest citations. The 10 most impactful research articles and the 10 most impactful review articles were listed in Appendix 3. The research article that received the highest number of citations was published in the *ChemMedChem* journal and discussed the chemical technique for the detection of fake medicines. The research article that received the highest rate of citations (number of citations per year) was published in Nanoscale journal and discussed the use of inkjet printing as an anti-counterfeiting strategy. The review article that received the highest number of citations was published in *The Lancet Infectious Disease* while the review article that received the highest rate of citations (number of citations per year) was published in *Expert Opinion on Drug Safety* journal and discussed the emerging technologies to combat fake medicines.

### Funding sponsors of the retrieved documents

Analysis of funding showed that 119 (12.2%) of the retrieved documents received financial funding. The main funding sponsor was the U.S. Department of Health and Human Services/ National Institutes of Health (n=14), followed by Wellcome Trust (n=12), the European Commission (n=11), and the National Natural Science Foundation of China (n-7).

## Discussion

The present study aimed to analyze global research publications on SF medical products using a bibliometric methodology. The growth of publications on SF medicines was influenced by the milestone initiatives by international organizations to fight falsified medicines [5]. The beginning of the second growth phase of publications coincides with the first international conference on counterfeit medicines launched in 1992 while the third phase coincides with the publication of the technical briefing by the *World Health Assembly* (WHA) in 2001. Furthermore, between 2001 and 2005 several international organizations including the WHO, *the International Federation of Pharmaceutical Manufacturers & Associations* (IFPMA), and *Pharmaciens Sans Frontières* held several round table meetings on counterfeit medicines. The overall growth of publications seen in the last decade was also attributed to the sale of fake medicines through online pharmacies [27, 28] and the trafficking of falsified medicines across borders in countries with regulatory policies in this regard [29, 30]. Researchers from the UK claimed that 25 % of surveyed general practitioners reported they had cared for individuals who had experienced adverse effects from medications ordered online [28].

The list of core journals has two journals, Lancet and BMJ, that are related to the public health domain. Falsified medicines have been described as a threat to public safety and health [31]. The spread of certain fake drugs used for malaria or cancer causes serious health, economic, and humanitarian harm [32, 33]. The use of falsified antimicrobial agents has serious public health implications including the rapid emergence of antimicrobial resistance with poor infection control [34]. The use of falsified or substandard cardiovascular drugs such as clopidogrel could increase the risk of morbidity and mortality in high-risk patients [35]. Another serious public health problem of SF medical products emerged with the spread of fake coronavirus vaccines in certain parts of the world [36, 37].

The productivity analysis for authors and countries indicated that research on SF medical products occurs in a large number of countries. The leading role of the US and certain European countries was not surprising since (1) it is in those countries where better infrastructures and more abundant resources for research are available [38]; (2) well-established and major pharmaceutical companies exist, and (3) falsified medicines is a global problem and present in developed countries as well as developing ones. India and China ranked 6th and 7th respectively in research productivity in this field. A report released by the Office of the United States Trade Representative (USTR) in 2019 claimed that India and China are the leading sources of falsified medicines globally [39]. The report stated that “up to 20 % of drugs sold in the Indian market are falsified and could represent a serious threat to patient health and safety”. However, representatives of the Indian pharmaceutical industry disagree with the US allegation and stated that up to 3.0 % of drugs in India were “not of standard quality”, and 0.02 % were spurious. Drug regulatory agency in India has implemented regulations specifically to fight falsified drugs [30]. China took several steps in the fight against falsified drugs especially after the death of more than a dozen infants in China due to fake milk powder that had little or no nutritional value [40]. China strengthened its regulatory policies after it joined the World Trade Organisation (WTO) in 2001. The WHO calculates that China’s State Drug Administration closed 1,300 illegal factories in 2003 alone [41].

Falsification of medicines is an old phenomenon [42] and affects people in all income categories. However, the problem tends to be more obvious in world regions with (1) intense cross-border movements, (2) high demand for less expensive drugs, and (3) weak national regulatory policies on the manufacturing and marketing of medications. One example is the spread of low-quality antimalarials in the Greater Mekong Subregion. Reports indicated that the use of falsified and substandard antimalarials in the Mekong region led to artemisinin resistance, creating new challenges for the eradication of malaria [43–45]. The problem of substandard and low-quality antimalarial drugs was noted by the WHO and experts from the Lao-Oxford-Mahosot Hospital Wellcome Trust Research Unit (LOMWRU) [46, 47].

Except for collaboration between researchers in the US and the UK with researchers in the Mekong region (Laos, Thailand, and Cambodia), the present study showed limited research collaboration at the author and cross-country levels. International research collaboration is considered one potential mechanism for enhancing the social and scientific relationship between countries [48]. The research collaboration with countries in the Mekong region was based on the problem of border malaria and the urgency of eliminating artemisinin-resistant *P. falciparum* before they spread in other parts of the world, specifically Africa [49]. One approach by the WHO to eliminate malaria from the Mekong area by 2030 depends on fighting cross-border trafficking of substandard and falsified antimalarial drugs. In the present study, research collaboration was observed between France and certain countries in Africa including Benin, Congo, and Burkina Faso. Similar research Collaboration existed between the UK and both Nigeria and Ghana. Falsified medicines are present in many African countries [9, 50] and the prevalence of substandard antimalarials was reported to be as high as 88.4 % in Africa region compared with 53 % in the South-East Asian region [51]. In most cases of falsified medicines in these regions, the problem was in low amounts of active pharmaceutical ingredients [52]. The research collaboration with countries in the South-East Asia region was stronger than that with African countries possibly because of the serious threat of the emergence of anti-malarial drug resistance in the Mekong region [53, 54].

Analysis of keyword co-occurrences indicated that falsified antimalarial drugs constituted a separate research topic. This was expected given that malaria is a major public health threat with more than 200 million affected people in Africa and the South-East Asian region [55]. Another research topic was sexual stimulants; sildenafil (Viagra®). A study found that 77 % of shipped Viagra ® by internet sites claiming to sell original Viagra® were substandard or fake. The same study reported that counterfeit Viagra usually came from non-U.S. addresses and had 30–50 % of the labeled active pharmaceutical ingredient [56]. Websites and social media are new powerful instruments to sell fake medicines and generate profit [57]. A large number of publications on counterfeit Viagra and other sexual stimulants were dedicated to the development of detection technologies [29]. It has been suggested to use blockchain technology as well as strengthening regulatory bodies and policies to fight the internet pharmaceutical trade [58]. The use of detection technology such as chromatography and spectrophotometry constituted another research topic in the retrieved documents. Such technologies are in use to detect falsified medications, especially in developing countries [29].

Collaboration between governments and the pharmaceutical industry is needed in the fight against falsified medicines. It has been argued that several countries and pharmaceutical companies tend to hide news about fake drugs to avoid national panic or because of their inability to fight the phenomenon [59]. Strengthening the role of pharmacovigilance centers is also needed in the coordinated fight against falsified medicines. When considering adverse drug reactions, pharmacovigilance personnel need to consider the source of the drug especially the internet sources.

### Policy implications of the current study

The current study has several implications on national and international health policy that are summarized below in 10 points.

1. Research activity on SF medical products enables policymakers and public health experts to quantify the problem and assess its impact on national health security. Furthermore, research on SF medical products allows governments to build anti-counterfeiting strategies based on the types of SF products and the stage at which these products enter the pharmaceutical supply chain.
2. One important point to strengthen research about SF products is to initiate a national pharmacovigilance center to receive reports about unexpected or low-quality medications. The diethylene glycol poisoning of children in Nigeria is a good example of how reporting and tracing of adulterated chemicals helped uncover the story of the adulterated paracetamol syrup [60, 61]. Public health experts need to take into consideration the presence and the spread of SF products to understand and fight global public health problems such as antimicrobial resistance. Lack of therapeutic benefit and resistance related to antimalarial drugs indeed caught the attention of many researchers. However, other falsified antimicrobial agents are present in different parts of the world but are either unnoticed or under-researched. Therefore, the authenticity of all types of antimicrobial agents needs to be periodically screened and tested to confirm good quality and the absence of any falsified antimicrobial agents [62].
3. The pharmaceutical industry should be fully involved in the fight against falsified drugs. One reason for the widespread presence of SF drugs is the high prices of certain groups of medications [63]. The pharmaceutical industry should take the initiative to make their prices more affordable especially in low- and middle-income countries. The pharmaceutical industry should also launch awareness campaigns to increase the knowledge of the public and those in the healthcare system about the presence of SF products and their health risks.
4. The search on SF products in the past few decades focused on antimicrobial medications and lifestyle medications such as sexual stimulants. However, this does not mean that there are no SF medications used for non-communicable diseases such as anti-cancer drugs and biological drugs. Future research and screening should include all categories of expensive and life-saving drugs. Counterfeit bevacizumab was present in different countries including the US and India [64, 65].
5. The finding that no research or insignificant research activity was present in different parts of the world such as the African region and the Eastern Mediterranean region does not mean that the problem of SF products in these regions is absent. Lack of research on SF products mostly suggests the lack of data or research capacity or absence of scholars interested in this field. The opposite is true. Countries with high research activity on SF products are not necessarily suffering from a widespread problem of SF products. Most of the research on SF products in high-income countries is dedicated to developing and implementing anti-counterfeiting policies, regulations, and technologies. In this regard, countries with reported incidents of falsified medications should develop regulations and strict laws to fight criminals involved in the manufacturing and marketing of SF products. Countries also need to invest in research and technologies needed to fight falsified medicines. Encouraging research in this field needs funding and training. This is an important area of research collaboration between high-income countries and resource-limited countries with a high burden of diseases and the potential presence of SF products. Investment of high-income countries in such collaboration will reflect positively on the national health of these countries because the widespread presence of SF products has an international dimension and could negatively affect international health security.
6. Research on SF medical products is of importance to pharmacists, physicians, nurses, public health experts, and policymakers. Therefore, editors of journals in these medical fields should take the initiative and launch a special call for papers on SF products to increase and stimulate the interest of scholars and professionals to write in this field. Public health journals have a large and diverse number of readers and publishing documents on SF products in these journals will have a positive impact on the fight against falsified drugs.
7. Comparative research on the regulations and laws implemented on criminals involved in the SF products is important to unify the punishment across different countries. Criminals in the field of SF products should not feel secure in any country and should always be held accountable for the potential loss of lives due to these products. Strict and tough punishments should be implemented across all countries on criminals manufacturing and marketing SF products.
8. Research on the success of various policies implemented by different high-income countries needs to be published to assess the success rate and effectiveness of each policy or group of policies and anti-counterweighting strategies.
9. Online pharmacies sell falsified medications and can be accessed through an internet connection. Online sales and online advertisements of medications through pop-up windows in social media need better monitoring and control by individuals and the healthcare system of the country. Websites responsible for the online sale of prescription medications need to be periodically verified and reviewed.
10. There are several new technologies developed for faster and accurate screening and detection of falsified medical products. Investment in the new and updated technologies and research on the accuracy and efficacy of the different technologies implemented in different countries for detection of falsified medications.

### Limitation

Scopus is a large database, but there are many unindexed health-related journals in Africa, Middle East, South-East Asia, Latin America, and East Europe. This created some sort of bias toward countries with journals indexed in Scopus or countries with English publications. Therefore, research productivity from other world regions might be under-estimated.

## Conclusions

The current study aimed to analyze and visualize documents published in a peer-reviewed journal on SF medical products. The key findings of the current bibliometric and visualization study are summarized as follows: (a) there was a steep growth of publications seen after the year 2001; (b) the publications were disseminated through a large number of journals, especially those in the field of pharmacy, public health, and chemistry; (c) authors involved in publishing the retrieved documents were affiliated with a relatively large number of countries, especially the USA and other English-speaking countries; (d) Cross-country collaboration was observed between developed countries and countries in the Mekong region and Africa; (e) active authors have limited interaction between them; and (f) falsified antimalarials and sexual stimulant medications were major therapeutic classes encountered in the retrieved documents. The current study is meant to stimulate researchers and academics, especially in developing countries, to get involved in this field through research investigation to identify falsified medications and their illegal manufacturing and marketing. Furthermore, pharmacists, nurses, and physicians need to get involved and report suspected medications to authorities to take firm action. The author of the current study recommends the following to endorse the safety of patients and global health security. First, research from regions with low research activity is needed and must be encouraged and funded to identify the size of the problem and the types of the therapeutic classes being marketed as falsified drugs. Second, high-income countries need to support other world regions in terms of building research capacities, training, and funding for scholars in low- and middle-income countries. Third, research on medications other than antimicrobials is needed especially anti-cancer drugs, biological drugs, and medications used in cardiovascular diseases. Fourth, there is an urgent need for international collaboration in implementing new updated technologies in the detection of SF products. This requires more research on the most effective methods and technologies. Research on the levels of awareness and extent of participation of pharmacists, nurses, and physicians on the anti-counterfeiting strategies is needed to have a comprehensive national policy in this field. Fifth, all healthcare providers need to be part of the war against SF products by reporting to pharmacovigilance centers about unexpected adverse drug reactions or failure to get the optimum therapeutic outcomes from certain well-known drugs. Finally, as a future recommendation for policymakers, harsh penalties need to be implemented on criminals involved in SF manufacturing and sale.

## Supplementary Information

**Additional file 1.**

**Additional file 2.**

## Data Availability

all data presented in this manuscript are available on the Scopus database using the search query listed in the methodology section.

## Abbreviations

SF: Substandard and falsified
WHO: World Health Organization
