# Supplementary material for: Substandard and falsified medical products: bibliometric analysis and mapping of scientific research
Source: Global Health. 2021 Sep 23;17:114. doi: 10.1186/s12992-021-00766-5 (PMC8460181; doi:10.1186/s12992-021-00766-5)
Supplement: Supplementary file 1 — Additional file 1. [file 12992_2021_766_MOESM1_ESM.docx]

**Appendix 1: Terms and phrases used to retrieve the relevant documents on SF products**

| **Step** | **Keyword** | **Number of documents** |
| --- | --- | --- |
| **1** | title (fake or substandard or falsified or *counterfeit or "drug fraud" or "medication fraud" or "spurious medicine*" or falsification or counterfeiting or "low quality" or "poor quality") w/2 ( medication or drug or medicine or pharmaceutical or antimicrobial or antimalarial or antibiotic or antiinfective or anticancer or "medicinal" or pharma*) | **1077** |
| **2** | title-abs ( fake or counterfeit* or substandard) and title-abs ( drug or medical or medicine or health) or title ( "poor-quality medicines") ) and all ( "falsified") and srctitle ( pharm* or drug ) and all ( "fake drug" or "counterfeit drug") | **103** |
| **3** | title ("pharma* fraud")  or  title ("falsified medical product*"  or  "counterfeit drugs") | **279** |
| **4** | title-abs (bogus  and  counterfeit)  or  title-abs ("low quality drug*"  and  counterfeit)  or  title-abs ("substandard and falsified"  and  drug* )  or  title ( "adulterated medicines")  and  all (drug  or  medication*  or  medicines) | **48** |
| **Total** | **#1 OR #2 OR #3 OR #4** | **1164** |

The search query in each step included quotation marks which were used to retrieve the exact phrase while the truncation with the asterisk was used as a wild card to retrieve any possible term. The Boolean operator (W/2) used in the first step means the keywords must be within two words as a maximum distance.

**Appendix 2: Search strategy implemented in the study on SF products**

**EXCLUDE false-positive documents**

**EXCLUDE title (cubebs or "data falsification" or "fish oil")**

**= 1161**

**Limit to documents published in peer-reviewed journals**

**= 997**

**Exclude Errata documents and articles in press (unpublished yet)**

**= 978**

**Exclude documents published in 2021**

**= 1126**

**Terms and phrases related to SF products**

**(Appendix 1)**

**= 1164**
