# Supplementary material for: Substandard and falsified medical products: bibliometric analysis and mapping of scientific research
Source: Global Health. 2021 Sep 23;17:114. doi: 10.1186/s12992-021-00766-5 (PMC8460181; doi:10.1186/s12992-021-00766-5)
Supplement: Supplementary file 2 — Additional file 2. [file 12992_2021_766_MOESM2_ESM.docx]

**Appendix 3 (A)** Top 10 most impactful research articles on substandard and falsified medical products [1-10]

| **Rate of citations*** | **Type of document** | **Number of citations** | **Journal name** | **Year** | **Title** | **Authors** |
| --- | --- | --- | --- | --- | --- | --- |
| 12.4 | Article | 174 | *ChemMedChem* | 2006 | *Characterization of solid counterfeit drug samples by desorption electrospray ionization and direct-analysis-in-real-time coupled to time-of-flight mass spectrometry* | Fernández, F.M., Cody, R.B., Green, M.D., Hampton, C.Y., McGready, R., Sengaloundeth, S., White, N.J., Newton, P.N. |
| 6.3 | Article | 146 | *Tropical Medicine and International Health* | 1997 | *Assessment of the incidence of substandard drugs in developing countries* | Shakoor, O., Taylor, R.B., Behrens, R.H. |
| 19.4 | Article | 136 | *BMJ Open* | 2013 | *Substandard and counterfeit medicines: A systematic review of the literature* | Almuzaini, T., Choonara, I., Sammons, H. |
| 8.7 | Article | 131 | *Analytica Chimica Acta* | 2005 | *NIR spectrometry for counterfeit drug detection: A feasibility study* | Rodionova, O.Ye., Houmøller, L.P., Pomerantsev, A.L., Geladi, P., Burger, J., Dorofeyev, V.L., Arzamastsev, A.P. |
| 32.3 | Article | 129 | *Nanoscale* | 2016 | *Three-dimensional quick response code based on inkjet printing of upconversion fluorescent nanoparticles for drug anti-counterfeiting* | You, M., Lin, M., Wang, S., Wang, X., Zhang, G., Hong, Y., Dong, Y., Jin, G., Xu, F. |
| 14.1 | Article | 127 | *Journal of Pharmaceutical Sciences* | 2011 | *The global counterfeit drug trade: Patient safety and public health risks* | Mackey, T.K., Liang, B.A. |
| 9.6 | Article | 125 | *Analytical Chemistry* | 2007 | *Reactive desorption electrospray ionization linear ion trap mass spectrometry of latest-generation counterfeit antimalarials via noncovalent complex formation* | Nyadong, L., Green, M.D., De Jesus, V.R., Newton, P.N., Fernández, F.M. |
| 6.2 | Article | 117 | *Analyst* | 2001 | *Identification of counterfeit drugs using near-infrared spectroscopy* | Henrique Frasson Scafi, S., Pasquini, C. |
| 14.4 | Article | 115 | *Advanced Materials* | 2012 | *Lithographically encoded polymer microtaggant using high-capacity and error-correctable QR Code for anti-counterfeiting of drugs* | Han, S., Bae, H.J., Kim, J., Shin, S., Choi, S.-E., Lee, S.H., Kwon, S., Park, W. |
| 17.7 | Article | 106 | *British Journal of Clinical Pharmacology* | 2014 | *Substandard drugs: A potential crisis for public health* | British Journal of Clinical Pharmacology |

**The rate of citations** is calculated by dividing the total number of citations received by the time elapsed since the publication of the article

**Appendix 3B** Top 10 most impactful review articles on substandard and falsified medical products [11-20]

| **Rate of citations*** | **Type of document** | **Number of citations** | **Journal name** | **Year** | **Title** | **Authors** |
| --- | --- | --- | --- | --- | --- | --- |
| 18.1 | Review | 253 | *Lancet Infectious Diseases* | 2006 | Counterfeit anti-infective drugs | Newton, P.N., Green, M.D., Fernández, F.M., Day, N.P., White, N.J. |
| 31.5 | Review | 252 | *The Lancet Infectious Diseases* | 2012 | Poor-quality antimalarial drugs in southeast Asia and sub-Saharan Africa | Nayyar, G.M.L., Breman, J.G., Newton, P.N., Herrington, J. |
| 13.9 | Review | 208 | *PLoS Medicine* | 2005 | The global threat of counterfeit drugs: Why industry and governments must communicate the dangers | Cockburn, R., Newton, P.N., Agyarko, E.K., Akunyili, D., White, N.J. |
| 11.1 | Review | 177 | *Tropical Medicine and International Health* | 2004 | Fake antimalarials in Southeast Asia are a major impediment to malaria control: Multinational cross-sectional survey on the prevalence of fake antimalarials | Dondorp, A.M., Newton, P.N., Mayxay, M., Van Damme, W., Smithuis, F.M., Yeung, S., Petit, A., Lynam, A.J., Johnson, A., Hien, T.T., McGready, R., Farrar, J.J., Looareesuwan, S., Day, N.P.J., Green, M.D., White, N.J. |
| 14.1 | Review | 169 | *Tropical Medicine and International Health* | 2008 | Substandard medicines in resource-poor settings: A problem that can no longer be ignored | Caudron, J.-M., Ford, N., Henkens, M., Macé, C., Kiddle-Monroe, R., Pinel, J. |
| 11.0 | Review | 165 | *Analyst* | 2005 | Pharmaceutical counterfeiting | Deisingh, A.K. |
| 10.8 | Review | 141 | *Journal of Antimicrobial Chemotherapy* | 2007 | Counterfeit or substandard antimicrobial drugs: A review of the scientific evidence | Kelesidis, T., Kelesidis, I., Rafailidis, P.I., Falagas, M.E. |
| 19.2 | Review | 115 | *Journal of Pharmaceutical and Biomedical Analysis* | 2014 | Understanding and fighting the medicine counterfeit market | Dégardin, K., Roggo, Y., Margot, P. |
| 36.0 | Review | 108 | *Expert Opinion on Drug Safety* | 2017 | A review of existing and emerging digital technologies to combat the global trade in fake medicines | Mackey, T.K., Nayyar, G. |
| 7.3 | Review | 73 | *Analytical and Bioanalytical Chemistry* | 2010 | Counterfeit drugs: Analytical techniques for their identification | Martino, R., Malet-Martino, M., Gilard, V., Balayssac, S. |

**References**

1. Almuzaini T, Choonara I, Sammons H: **Substandard and counterfeit medicines: A systematic review of the literature**. *BMJ Open* 2013, **3**(8).doi:10.1136/bmjopen-2013-002923

2. Fernández FM, Cody RB, Green MD, Hampton CY, McGready R, Sengaloundeth S, White NJ, Newton PN: **Characterization of solid counterfeit drug samples by desorption electrospray ionization and direct-analysis-in-real-time coupled to time-of-flight mass spectrometry**. *ChemMedChem* 2006, **1**(7):702-705.doi:10.1002/cmdc.200600041

3. Han S, Bae HJ, Kim J, Shin S, Choi SE, Lee SH, Kwon S, Park W: **Lithographically encoded polymer microtaggant using high-capacity and error-correctable QR Code for anti-counterfeiting of drugs**. *Advanced Materials* 2012, **24**(44):5924-5929.doi:10.1002/adma.201201486

4. Henrique Frasson Scafi S, Pasquini C: **Identification of counterfeit drugs using near-infrared spectroscopy**. *Analyst* 2001, **126**(12):2218-2224.doi:10.1039/b106744n

5. Johnston A, Holt DW: **Substandard drugs: A potential crisis for public health**. *British Journal of Clinical Pharmacology* 2014, **78**(2):218-243.doi:10.1111/bcp.12298

6. Mackey TK, Liang BA: **The global counterfeit drug trade: Patient safety and public health risks**. *Journal of Pharmaceutical Sciences* 2011, **100**(11):4571-4579.doi:10.1002/jps.22679

7. Nyadong L, Green MD, De Jesus VR, Newton PN, Fernández FM: **Reactive desorption electrospray ionization linear ion trap mass spectrometry of latest-generation counterfeit antimalarials via noncovalent complex formation**. *Analytical Chemistry* 2007, **79**(5):2150-2157.doi:10.1021/ac062205h

8. Rodionova OY, Houmøller LP, Pomerantsev AL, Geladi P, Burger J, Dorofeyev VL, Arzamastsev AP: **NIR spectrometry for counterfeit drug detection: A feasibility study**. *Analytica Chimica Acta* 2005, **549**(1-2):151-158.doi:10.1016/j.aca.2005.06.018

9. Shakoor O, Taylor RB, Behrens RH: **Assessment of the incidence of substandard drugs in developing countries**. *Tropical Medicine and International Health* 1997, **2**(9):839-845.doi:10.1046/j.1365-3156.1997.d01-403.x

10. You M, Lin M, Wang S, Wang X, Zhang G, Hong Y, Dong Y, Jin G, Xu F: **Three-dimensional quick response code based on inkjet printing of upconversion fluorescent nanoparticles for drug anti-counterfeiting**. *Nanoscale* 2016, **8**(19):10096-10104.doi:10.1039/c6nr01353h

11. Caudron JM, Ford N, Henkens M, Macé C, Kiddle-Monroe R, Pinel J: **Substandard medicines in resource-poor settings: A problem that can no longer be ignored**. *Tropical Medicine and International Health* 2008, **13**(8):1062-1072.doi:10.1111/j.1365-3156.2008.02106.x

12. Cockburn R, Newton PN, Agyarko EK, Akunyili D, White NJ: **The global threat of counterfeit drugs: Why industry and governments must communicate the dangers**. *PLoS Medicine* 2005, **2**(4):0302-0308.doi:10.1371/journal.pmed.0020100

13. Dégardin K, Roggo Y, Margot P: **Understanding and fighting the medicine counterfeit market**. *Journal of Pharmaceutical and Biomedical Analysis* 2014, **87**:167-175.doi:10.1016/j.jpba.2013.01.009

14. Deisingh AK: **Pharmaceutical counterfeiting**. *Analyst* 2005, **130**(3):271-279.doi:10.1039/b407759h

15. Dondorp AM, Newton PN, Mayxay M, Van Damme W, Smithuis FM, Yeung S, Petit A, Lynam AJ, Johnson A, Hien TT *et al*: **Fake antimalarials in Southeast Asia are a major impediment to malaria control: Multinational cross-sectional survey on the prevalence of fake antimalarials**. *Tropical Medicine and International Health* 2004, **9**(12):1241-1246.doi:10.1111/j.1365-3156.2004.01342.x

16. Kelesidis T, Kelesidis I, Rafailidis PI, Falagas ME: **Counterfeit or substandard antimicrobial drugs: A review of the scientific evidence**. *Journal of Antimicrobial Chemotherapy* 2007, **60**(2):214-236.doi:10.1093/jac/dkm109

17. Mackey TK, Nayyar G: **A review of existing and emerging digital technologies to combat the global trade in fake medicines**. *Expert Opinion on Drug Safety* 2017, **16**(5):587-602.doi:10.1080/14740338.2017.1313227

18. Martino R, Malet-Martino M, Gilard V, Balayssac S: **Counterfeit drugs: Analytical techniques for their identification**. *Analytical and Bioanalytical Chemistry* 2010, **398**(1):77-92.doi:10.1007/s00216-010-3748-y

19. Nayyar GML, Breman JG, Newton PN, Herrington J: **Poor-quality antimalarial drugs in southeast Asia and sub-Saharan Africa**. *The Lancet Infectious Diseases* 2012, **12**(6):488-496.doi:10.1016/S1473-3099(12)70064-6

20. Newton PN, Green MD, Fernández FM, Day NP, White NJ: **Counterfeit anti-infective drugs**. *Lancet Infectious Diseases* 2006, **6**(9):602-613.doi:10.1016/S1473-3099(06)70581-3
